# Survival outcomes and response rates among patients with recurrent or metastatic head and neck squamous cell carcinoma

**DOI:** 10.6026/973206300210044

**Published:** 2025-01-31

**Authors:** Soumya Allurkar, Hiren Hansraj Patadiya, Himani Marmat, Debapriya Kundu, Satinder Pal Singh Tulsi, Satya Prakash Gupta

**Affiliations:** 1 Department Oral and Maxillofacial Surgery, PMNM Dental College and Hospital, Bagalkot, Karnataka, India; 2 Dental Practice, My Dental Southbridge, 305 Main st Southbridge, MA, USA-01550; 3 Department of Medicine, Government Medical College, Ratlam, Madhya Pradesh, India; 4 Intern, Kalinga Institute of Dental Sciences, KIIT Deemed to be University, Patia - 751024, Odisha, India; 5 Department of Oral and Maxillofacial Surgery, Chandra Dental College, Safedabad, Barabanki, Lucknow, Uttar Pradesh, India

**Keywords:** Checkpoint inhibitors, head and neck squamous cell carcinoma (HNSCC), recurrent, metastatic

## Abstract

Evaluation of survival outcomes and response rates among patients with recurrent or metastatic head and neck squamous cell carcinoma (HNSCC) treated with checkpoint inhibitors is of interest. Data regarding overall survival (OS), progression free survival (PFS), response rate, PD-L1 combined positive score (CPS) was retrieved. A total of 412 patients with histo-pathologically confirmed recurrent or metastatic HNSCC who received checkpoint inhibitor (CPI) treatment were ultimately included as members of the cohort. The median overall survival was 13.1 months. Median PFS was 4.1 months. The estimated 1-year overall survival was 53.9% while estimated 1-year PFS was 9.7%. Thus, the use of CPI therapies for first - or second-line treatment of recurrent or metastatic head and neck squamous cell carcinoma is shown.

## Background:

The immune system naturally contains immunological checkpoints. Their function is to keep cells that are healthy in the body from being destroyed by an overly powerful immune response [1, 2- 3]. When T cell surface proteins identify and attach to complementary proteins on the other cells, including some tumor cells, immunological checkpoints are activated. Authors refer to these proteins as immunological checkpoint proteins [4, 5-6]. The T cells receive an "off" signal when the partner and checkpoint proteins join together. This may stop the cancer from being destroyed by the immune system [5, 6-7]. Immuno-checkpoint inhibitors are immunotherapy medications that prevent checkpoint proteins from attaching to their companion proteins. By doing this, the "off" signal is not sent, enabling T lymphocytes to destroy cancer cells [8, 9-10]. Historically, patients with "recurrent or metastatic head and neck squamous cell carcinoma" (HNSCC) have had a poor prognosis and few viable therapeutic options if their illness progressed while they were undergoing platinum-based chemotherapy [11, 12- 13]. Checkpoint inhibitors (CPIs), either in combination or separately from chemotherapy, have been scientifically shown to have better results than conventional cytotoxic chemotherapy during the past few years and they are now considered mainstream recommended treatment for patients suffering "recurrent or metastatic HNSCC" [14, 15-16]. Nivolumab and Pembrolizumab, response rates are still poor, most patients do not benefit from CPI therapy and there are still concerns about the best clinical immunotherapy sequencing and selection for different patient subgroups with HNSCC [17, 18- 19]. In a study, the "overall response rate" was higher among HPV-positive patients. However, a recent meta-analysis of trials examining CPI therapy in HNSCC did not show significant variations in rates of progressing or stable illness comparing HPV-positive with HPV-negative patients [20, 21- 22]. Results for patients being administered CPI for "recurrent or metastatic HNSCC" in study settings other than clinical trials are scarce. Only individuals administered with nivolumab in the second-line situation have been incorporated in the results of a multidisciplinary cohort of 88 individuals suffering from "recurrent or metastatic HNSCC" who received immunotherapy, according to a study [23, 24]. In that trial, poorer OS and PFS were linked to an "Eastern Cooperative Oncology Group" (ECOG) rating of performance of 2 or 3.T-cell infiltration, immune cell activation and T-cell-inflamed gene expression have all been implicated in the enhanced efficacy of checkpoint inhibitor therapy for HPV-associated malignancies [12, 13, 14-15]. While one study found that individuals with HPV-positive tumors had a higher overall response rate to CPI therapy than patients with HPV-negative malignancies, another study found no discernible variation in response by HPV status [11, 12, 13-14]. Patients in the general public who are managed in clinical practice are frequently older, exhibit poorer ECOG performance status and suffer from worse symptoms of disease and/or concomitant medical disorders, in contrast to patients participating in clinical trials, who must satisfy strict inclusion or exclusion criteria. Consequently, there is limited universality of trial results to the practical situation [20, 21, 22, 23- 24]. Therefore, it is of interest to evaluate survival outcomes and response rates among patients with recurrent or metastatic head and neck squamous cell carcinoma treated with checkpoint inhibitors.

## Methods and Materials:

## Study design and participants:

A retrospective analysis of medical records was conducted on patients having histologically or cytologically proven HNSCC who had CPI therapy in reoccurring or metastatic illness at a single tertiary care facility. Researchers included patients over the age of 18 who had been administered pembrolizumab or nivolumab alone or in conjunction with chemotherapy for oral cancer, larynx cancer, oropharynx cancer, or hypopharynx cancer. This study excluded patients who were treated with immunotherapy for "head and neck basal cell carcinoma", "cutaneous squamous cell carcinoma", or "melanoma". Additionally excluded were those on pembrolizumab in a clinical study for patients with an elevated likelihood of recurrence (but no confirmed recurrence). Overall survival and PFS were defined from CPI therapy initiation until the date of death from any cause for OS and until the date of radiographic disease progression or death for PFS. Radiographic response was defined as best overall response on subsequent radiographic assessment after CPI initiation as documented in radiology reports and progress notes. Patients with complete or partial radiographic response were categorized as responders, whereas patients with stable disease, progressive disease, or mixed response (discordant shrinkage and progression in separate lesions) were categorized as non-responders. A PD-L1 test uses a sample of cancerous tumor tissue to measure how much of a protein called PD-L1 is found on the cancer cells. If anyone has certain types of cancer, PD-L1 testing can check whether you may benefit from a type of cancer treatment called immunotherapy. Immunotherapy helps one own immune system fight cancer. Normally, PD-L1 is found on certain healthy cells. It acts as a kind of brake to stop cells in your immune system, called T cells, from attacking healthy cells in body. If cancer cells have high amounts of PD-L1, they can turn your T cells off so they can't attack the cancer cells. If high amounts of PD-L1 are found on cancer cells, immunotherapy medicines called immune checkpoint inhibitors may be used. These medicines prevent the PD-L1 protein from putting the brakes on T cells. This frees T cells to fight cancer. Immunotherapy can help stop or slow the growth of many types of cancers that have PD-L1. Immunotherapy has fewer side effects than cancer chemotherapy. But it can cause serious side effects in some people and not everyone benefits from it. Data regarding overall survival (OS), progression free survival (PFS), response rate, PD-L1 combined positive score (CPS) was retrieved.

## Statistical analysis:

For all statistical analyses, we utilized Stata, version 16.1 (StataCorp LLC). "Overall survival and PFS were defined from CPI therapy initiation until the date of death from any cause for OS and until the date of radiographic disease progression or death for PFS." In addition to estimating the median OS and PFS using Kaplan-Meier methods, hazard ratios (HRs) and 95% CIs related to important covariates were estimated using multivariable Cox proportional hazards regression.

## Results:

412 patients diagnosed histo-pathologically with recurrent or metastatic HNSCC who were treated with CPI were finally included members of cohort in this cohort study. 216 (52.42%) patients were found to have distant metastatic disease. 150 (36.4%) patients were found to have Unresectable locoregional recurrence only. 46 (11.16%) patients had both distant metastatic and unresectable locoregional recurrence. In 246 (59.4%) patients CPI was first-line systemic therapy, while in 166 (40.6%) patients, CPI was second-line systemic therapy. Pembrolizumab monotherapy was most common CPI administered in 316 (76.69%) patients being followed by Nivolumab monotherapy in 54 (13.1%) patients and Pembrolizumab with chemotherapy in 42 (10.19%) (Table 1). In our study median OS was 13.1 months. Median PFS was 4.1 months. The estimated 1-year OS was 53.9% while estimated 1-year PFS was 9.7% (Table 2). The overall response rate with CPI was 31.2%. 8.3% patients showed complete response. 22.1% patients showed partial response, 9.6% showed mixed response. 20.7% patients had stable disease while 40.8% patients had disease progression (Table 3). The overall response rate was greater. The PD-L1 results were obtained for 186 patients. PD-L1 combined positive score (CPS) combined positive score (CPS) findings has been evaluated. It showed that median OS, PFS and overall response rate was greater when CPS <1 as compared to CPS ≥1. Similarly, median OS, median PFS and overall response rate was greater when CPS <20 as compared to CPS ≥20 (Table 4).

## Discussion:

If "recurrent or metastatic head and neck squamous cell carcinoma" (HNSCC) worsened while receiving platinum-based chemotherapy, patients historically had a poor prognosis and few effective treatment choices [6, 7, 8-9]. CPIs are now widely recommended as a treatment for patients with "recurrent or metastatic HNSCC" after scientific studies in recent years have demonstrated that they outperform traditional cytotoxic chemotherapy, either in combination or alone. Nivolumab, Pembrolizumab and Pembrolizumab are some of the CPIs [17, 18- 19]. The majority of patients do not benefit from CPI therapy, response rates remain low and questions remain regarding the optimal clinical immunotherapy sequencing and selection for various patient categories with HNSCC. In our study median OS was 13.1 months in patients treated with CPI. The findings are similar to the finding of other clinical trials where the median OS was between 11.5 months and 13 months. In our study mean PFS was 4.1 months. The findings are similar to the findings of other clinical trials where the mean PFS was 4.0 months to 4.8 months [13, 14, 15-16]. Immunological checkpoints are found in the immune system by nature. Their role is to prevent the body's healthy cells from being killed by an over reactive immune response [25-26]. Immunological checkpoints are triggered when T cell surface proteins recognize and bind to complementary proteins on other cells, including some tumor cells. These proteins are known as immunological checkpoint proteins by the authors [14, 15- 16]. When the partner and checkpoint proteins bind together, the T cells get a "off" signal. This could prevent the immune system from destroying the malignancy [15, 16- 17]. Immunotherapy drugs known as immuno-check point inhibitors stop checkpoint proteins from binding to their companion proteins and this prevents the "off" signal from being sent, allowing T lymphocytes to eliminate cancer cells [18, 19-20]. The overall response rate with CPI was 31.2%.The findings of present trial are similar to the findings of other clinical trial where overall response rate was between 30.0% and 35% [24, 25, 26-27]. The immune system naturally contains immunological checkpoints. Patients with HPV had a greater "overall response rate" in one research. However, when comparing HPV-positive and HPV-negative patients, a recent meta-analysis of trials looking at CPI therapy in HNSCC did not find any appreciable differences in rates of stable or advancing illness [26-27]. There are few results for patients receiving CPI for "recurrent or metastatic HNSCC" in research contexts other than clinical trials. According to study, the results of a multidisciplinary cohort of 88 patients with "recurrent or metastatic HNSCC" who received immunotherapy only include those who were given nivolumab in the second-line scenario [13, 14, 15-16].

## Conclusion:

The use of checkpoint inhibitors therapies for first/second-line treatment of recurrent or metastatic head and neck squamous cell carcinoma is reported.

## Figures and Tables

**Table 1:** Basic details of study participants of cohort

	**N= 412**
Distant metastatic disease	216(52.42%)
Unresectable locoregional recurrence only	150 (36.4%)
Both distant metastatic and unresectable locoregional recurrence	46 (11.16%)
CI therapy as first-line systemic therapy	246 (59.4%)
CI therapy as second or later line	166 (40.6%)
Pembrolizumab monotherapy	316 (76.69%)
Nivolumab monotherapy	54 (13.1%)
pembrolizumab with chemotherapy	42 (10.19%)

**Table 2:** Details about OS and PFS

**Median OS**	**13.1 months**	**(IQR, 4.2-36.7 months)**	**Median OS**	**13.1 months**
Median PFS	4.1 months	(IQR, 2.0-18.1 months)	Median PFS	4.1 months
Estimated 1-year OS	53.90%	(95% CI, 45.6%-59.9%)	Estimated 1-year OS	53.90%

**Table 3:** Response rate with CPI

**Response rate with CPI**	**Rate**
Overall response rate	31.20%
Complete response rate	8.30%
Partial response rate	22.10%
Mixed response rate	9.60%
Stable disease rate	20.70%
Disease progression rate	40.80%

**Table 4:** PD-L1 combined positive score (CPS)

	**Median OS**	**Median PFS**	**Overall response**
CPS ≥1	13.0 months	4.7 months	37%
CPS <1	26.6 months	12.6 months	40%
	Log rank p=0.64	Log rank p=0.44	OR 0.87 (95% CI 0.28 - 2.74)
CPS ≥ 20	15	4	34%
CPS <20	18.8	6.4	39%
	Log rank p=0.53	Log rank p=0.66	OR 0.81 (95% CI 0.32 - 2.04)
